# Playground activities and gender variation in objectively measured physical activity intensity in Australian primary school children: a repeated measures study

**DOI:** 10.1186/s12889-018-6005-5

**Published:** 2018-09-10

**Authors:** Dean A. Dudley, Wayne G. Cotton, Louisa R. Peralta, Matthew Winslade

**Affiliations:** 1 Department of Educational Studies, Faculty of Education Studies, Macquarie University, 1 University Ave Macquarie University NSW, Sydney, 2109 Australia; 2 Sydney School of Education and Social Work, University of Sydney. University of Sydney NSW 2006, Sydney, Australia; 3 Sydney School of Education and Social Work, University of Sydney. University of Sydney NSW 2006, Sydney, Australia; 4 School of Teacher Education, Charles Sturt University. Panorama Ave Bathurst, Bathurst, NSW Australia

**Keywords:** Children, Health promotion, Playgrounds, New South Wales, Observational study

## Abstract

**Background:**

Recent studies have sought to address the limited time for physical activity by focusing on increasing physical activity intensity among students during non-curricula periods and specifically school break times. We objectively measured the intensity of student physical activity (PA) during recess and lunch breaks at primary schools in the Western Sydney region of New South Wales (NSW), Australia using a 12-month repeated measures observation design study.

**Methods:**

Systematic direct observation of recess and lunch breaks over a ten-week period in 2014 and 2015. 120 recess and lunch breaks across twenty schools (2014) with 839 periodic observations and across 15 schools with 587 periodic observations in 2015. Both observation periods were conducted over 10-weeks in Term 4 (September – December).

**Results:**

The mean proportion of vigorous physical activity reported as a percentage (%VPA) across both time points was 16.6% (SD = 23.4). 36.8% (SD = 26.0) of time was spent walking and the remaining time (46.6%; SD = 30.4) was spent in sedentary activities. There was a significant decline in %VPA and increase in sedentary activity (*p* < 0.01) between the two time periods of measurement. In 2014, boys spent twice as much time in %VPA than girls during breaks in the school day and in 2015 this increased to nearly three times as much time in %VPA. %VPA also varied on the type of surface PA took place and the types of activities the children were allowed to undertake during breaks.

**Conclusions:**

Recess and lunch breaks potentially offer an opportunity for children to participate in unstructured PA during the school day. Substantial variations in the %VPA during these periods exist. Addressing playground gender participation disparities and space usability/accessibility may be a necessary first step in promoting higher PA intensities during breaks.

## Background

Evidence acquired over the last decade in Australia alone clearly demonstrates the need for increased physical activity levels and physical activity intensity in primary school-aged children [1, 2]. Current research suggests that only about half of all Australian primary school aged children are meeting the National Physical Activity and Sedentary Behaviour Recommendations [3, 4]. This is concerning considering the strong relationship between achieving the minimum of 60 min of physical activity each day and positive physical, social and mental health [5] and a range of academic outcomes, including cognition [6], on-task behaviour [7] and academic achievement [8].

Despite the availability of physical activity health promotion programs for primary schools, the Independent Sport Panel Report: The Future of Sport in Australia [9] and Auditor’s General’s report on Physical Activity in NSW Government Primary Schools [10] argue they are inadequate. Both report an insufficient allocation of school resources for children to be adequately active in terms of time and intensity (i.e. during physical education, school sport, and free play), limited teacher training in understanding the importance of physical activity and how to effectively motivate students to be active, and a lack of supportive resources for schools and primary teachers to encourage physical activity.

Recent systematic reviews have sought to examine the interventions focusing on increasing children’s physical activity levels during school break times [11, 12]. These reviews report that interventions based on playground markings and equipment do increase the physical activity of schoolchildren during school break times in the short to medium term. There have been an abundance of intervention studies seeking to increase student physical activity levels during recess and lunch break times, but there have been limited studies focusing on physical activity intensity, and to the authors’ knowledge no objective observational studies (free from intervention) describing the intensity of student physical activity participation over time in NSW primary schools. The primary aim of this observational study was to examine the playground activity type and gender-related variation in physical activity intensity among a large group of NSW primary school children. Additionally, we report on covariates such as surface type, temperature, school socio-economic status, and time of day to determine if these factors had any influence on physical activity intensity during recess and lunch breaks.

## Method

Researchers contacted and invited 40 primary schools from the Greater Western Sydney area of NSW, Australia. This area was chosen due to the prevalence of students in this region not meeting the physical activity guidelines^1^. The first twenty schools to respond to the invitation were recruited into the study as this was the financial capacity of the study. All schools were Grades K-6 with a student age range of 4–12 years.

The primary outcome variable for this study was the intensity of PA levels during recess and lunch breaks as a mean percentage of time available during those periods. Simply, we were most interested in mean proportion of students engaging in vigorous physical activity (%VPA), Walking (%Walking), or sedentary activity (%sedentary activity). This was measured using the System of Observing Physical Activity in Recreation and Communities (SOPARC) [13] with simultaneous observations conducted by two research assistants trained to the gold standard. Covariates included sex of student (captured by uniform observation), area type (hard or soft surface), activity type (captured using SOPARC coding of activity types), temperature (captured from the Bureau of Meteorology website), and school socio-economic status (Socio-Economic Indexes for Areas – SEIFA [14]; and Index of Community Socio-Educational Advantage – ICSEA [15]).

PA was measured using direct observation of three randomly selected recess and lunch breaks (which could range between 10 and 45 min) on three separate days from each school over a 10-week period in Term 4 (September – December) of 2014 and 2015. A repeated measures design was conducted in order to reduce school variability. No other natural experiments were being conducted in the schools that remained in the study during these two observation phases. The SOPARC [13] was used to collect these data and is based on momentary time sampling procedures in which orderly and recurrent scans of individuals and contextual factors within predetermined target areas are made. iPad tablets (Apple Inc., USA) installed with the iSOPARC Application Version 1.75 (CIAFEL, Portugal: http://ciafel.fade.up.pt) were used to provide instead of the traditional paper version of the instrument. The iSOPARC is a smart device application that implements the SOPARC protocol to generate data from field observations when used by trained observers. It provided the capacity to store, process and export data in a more timely and secure manner than afforded with paper-based versions of the instrument. The iSOPARC contains a digital counter that calculates the proportion of physical activity intensity as a percentage observed time (i.e. coded as %VPA, %walking, or %sedentary), the prominent activity type (i.e chasing, football, racquet games, etc.…), target area mapping, project-based data management, and cloud compatible data export functions.

Boundaries for the two most commonly utilised areas for student physical activity during recess and lunch periods (as identified by the school principal or their proxy) were marked on a school map and assessed for suitability for iSOPARC scanning in negotiation. One undercover and one exposed area were needed for each school. Observations were only made when it was not raining, thus when the weather permitted outdoor play. Ensure consistency with observation protocol, photo documentation of all the pre-determined settings (from each school) were taken by research staff and examined in order to classify the surface type as either hard (concrete or asphalt) or soft (grass or dirt).

Four research assistants were trained as iSOPARC observers and conducted the observations. On completion of the training, the observers were only allowed to commence the observations for this study when an interrater agreement of 85% or more on all variables on pre-recorded “gold-standard” DVDs and during live field practice was reached. Thirty-two field-based inter-rater reliability checks were conducted during the 10-week observation period. During reliability checks, two observers independently coded the same students in the same lunch or recess break.

In the traditional application of the iSOPARC tool, a scan of each subject is electronically coded and identified by: sex (male or female), intensity of activity (sedentary, walking, or very active), and whether they are a child, teen, adult or senior. During a scan of each subject, the physical activity of each individual was coded as sedentary (i.e., lying down, sitting, or standing), walking, or vigorous. The activity codes used in the SOPARC instrument have been validated by heart rate monitoring of youths from kindergarten through 12th grade^15,^ and by pedometry in primary school physical education classes^15^. Separate scans were made for females and males, and entries are also made for time of day, temperature, area accessibility, area usability and presence of supervision. Each observation was conducted twice during the recess and lunch breaks for both females and males (i.e. four observations in total for recess breaks and another four observations for lunch breaks). Additional data recorded prior to the direct observation scans included; temperature and UV level at the start and end of the observation period; whether the observation was made at recess or lunch; start and finish times of recess and lunch; and whether the area was shaded or not.

Socioeconomic status (SES) was calculated based on two Indexes (based on postcode of residence using Socio-Economic Indexes for Areas – SEIFA; and the school’s Index of Community Socio-Educational Advantage – ICSEA as determined by the Australian Curriculum, Assessment and Reporting Authority).

In an effort to minimise bias, inter-rater reliability checks on 4% of the iSOPARC observations were randomised in order to prevent possible collusion. Recess and lunch break observations were randomly selected and observers and schools were given limited notice of when a reliability check was going to occur (usually less than 24 h).

Ethics approval was obtained from an Australian University Human Research Ethics Committee and the NSW Department of Education. Written informed consent was provided by school principals.

Data analysis of the iSOPARC observations were analysed using Statistical Package for Social Science (SPSS) version 24. School playground physical activity was assigned as the dependent variable. Vigorous physical activity (VPA), walking and sedentary activity were the principal outcomes of interest as a mean proportion of total observations. Student sex, activity type playground surface type, temperature, SES and time of day were assigned as independent variables.

Measures of central tendency were calculated for iSOPARC data for the entire sample and then stratified by sex (boys and girls), school environment (shaded and unshaded areas) and activity type. The relationship between these covariates and activity intensity were calculated using Analysis of Covariance (ANCOVA) in SPSS version 21. The between group difference of boys and girls was calculated using an Analysis of Variance (ANOVA) in SPSS version 21.

Pearson product moment correlations were calculated on each observation for VPA based on school ICSEA, temperature (obtained via the Bureau of Meteorology website; see www.bom.gov.au), and time of day (accessed via iSOPARC). Individual schools were not included because of the variability in contextual factors in each of the different schools (i.e., population, total area, school policy around play spaces).

## Results

### Descriptive data

Table 1 provides an overview of the schools that consented to being in the study. The table shows that 55% of the schools in 2014 and 66% in 2015 were located in the 5th decile (highest) of the Socio-Economic Indexes for Areas (SEIFA), with the remaining schools being in the lower deciles. However, this measure is based on postcode of the school alone and may be a misleading proxy for socio-economic status (SES) of a school population. When the Australian Curriculum, Assessment and Reporting Authority’s Index of Community Socio-Educational Advantage (ICSEA) [15] is used, the analysis reveals around 30% of the students enrolled in the participating schools are in the bottom quarter of the Australian population for SES, with only 20% being in the top quarter. The ICSEA may be a more indicative SES proxy for school-based studies because it calculates students’ family backgrounds (parents’ occupation, school education and non-school education). In addition to these student-level factors, the school’s geographical location and the proportion of Indigenous students are considered when summarising educational advantage or disadvantage at the school level. ICSEA also provides a scale that numerically represents the relative magnitude of this influence, and is constructed taking into account both the student- and the school-level factors. Five schools were lost at the follow-up phase as they chose to participate in another study that could have potentially contaminated findings in the follow-up period.

**Table 1:** Demographic characteristics of the schools recruited in 2014 and 2015 of the study

		2014	2015
Schools stratified by SEIFA Index (% of schools) based on postcode of school			
	1	0 (0%)	0 (0%)
	2	1 (5%)	1 (7%)
	3	3 (15%)	1 (7%)
	4	5 (25%)	3 (20%)
	5	11 (55%)	10 (66%)
Distribution of school students stratified by the ICSEA			
	Bottom Quarter	30.6%	28.7%
	Middle Quarters	49.8%	51.1%
	Top Quarter	19.6%	20.2%

### Reliability of iSOPARC in primary school settings

Of the 60 field-based inter-rater reliability observation checks that were conducted, a high degree of reliability was found between measurements. The mean Intraclass Correlation Coefficient (ICC) for physical activity coding was .912 with a 95% confidence interval. The range of ICC was from .885 to .932 (F = 11.324, *p* < .001).

### Main results

During the study, we made 839 and 587 observations of student behaviour in the playground in 2014 and 2015, respectively. The fall in observations across the study was due a five schools being unavailable in 2015 as they were undertaking a separate intervention study that the researchers felt may have contaminated the results. In 2014, 425 observations occurred on hard surfaces and 414 on grass. In 2015, 284 observations occurred on hard surfaces and 302 were on grass. The mean proportion of time spent in the different intensities of PA during recess and lunch breaks can be found in Table 2. The mean %VPA across both time points was 16.6% (SD = 23.4). 36.8% (SD = 26.0) of time was spent walking and the remaining time (46.6%; SD = 30.4) was spent in sedentary activities.

**Table 2:** Mean proportion of student activity intensity (M%) and standard deviations (SD) during recess and lunch break in 2014 and 2015

Category	Recess & lunch behaviour (Mean proportion %)		Recess & lunch behaviour (Mean proportion %) Boys Obs.		Recess & lunch behaviour (Mean proportion %) Girls Obs.		Between group difference Boys and Girls	
	2014 M% (SD) (*n* = 839)	2015 M% (SD) (*n* = 587)	2014 M% (SD) (*n* = 420)	2015 M% (SD) (*n* = 295)	2014 M% (SD) (*n* = 419)	2015 M% (SD) (*n* = 292)	Mean Difference (*p*-value) 2014	Mean Difference (*p*-value) 2015
Intensity of Physical Activity (via SOPARC)								
Sedentary activities	43.5 (30.9)	50.9 (29.3)	38.4 (30.4)	44.0 (28.7)	48.6 (30.7)	58.0 (28.2)	10.2 (< 0.01)	14.0 (< 0.01)
Walking	37.8 (26.8)	35.2 (24.8)	37.6 (26.5)	35.8 (23.7)	38.0 (27.2)	35.2 (25.9)	−0.4 (0.41)	−0.6 (0.61)
Vigorous activities (VPA)	18.8 (25.0)	13.5 (20.5)	24.0 (27.1)	20.2 (24.2)	13.5 (21.5)	6.8 (12.9)	−10.5 (< 0.01)	−13.4 (< 0.01)
	Recess & lunch behaviour (Mean proportion VPA%)		Between group differences for hard and soft surface areas at baseline and follow-up					
Physical Activity observations (via SOPARC)	2014 M% (SD)	2015 M% (SD)	Mean Difference (p-value) 2014	Mean Difference (*p*-value) 2015				
Cement or Asphalt (*n* = 425 in 2014; *n* = 284 in 2015) (Hard surface)	12.7 (22.4)	5.7 (12.8)						
Grass (*n* = 414 in 2014; *n* = 302 in 2015) (Soft surface)	24.9 (26.4)	20.9 (23.5)	−12.2 (< 0.01)	−15.2 (< 0.01)				

Across the observation periods, boys were significantly more active than girls during recess and lunch with boys engaging in %VPA 22.4% (SD = 26.0; *p* < 0.01) of their recess and lunch breaks compared with girls who only spent 10.8% of recess and lunch break in %VPA (SD = 18.7). The percentage of time spent walking between boys and girls was comparable at 36.9% (SD = 25.4) and 36.8% (SD = 26.6), respectively. Girls spent a significantly larger percentage of their recess and lunch breaks in sedentary activities (p < 0.01) (52.5%; SD = 30.0), compared with boys (40.7%; SD = 29.8).

Primary school children spent more time being vigorously active on grassed areas compared with hard surfaced areas. In fact, grassed areas were more than twice as conducive to %VPA (23.2%; SD = 25.3), than concrete or asphalt covered surfaces (9.9%; SD = 19.1).

Table 3 reports the differences in activity based on sex and activity type. Students (as a whole) spent the most time being vigorously active during recess and lunch when playing rugby/touch football. However, the disparity between boys and girls in this activity could only be compared in 2014, as no girls were observed playing rugby/touch football at follow-up in any of the observed schools in 2015. More boys participated compared with girls in this type of activity (57 vs 14) and were more active (44.6%VPA vs 39.6%VPA) during the game. The results also indicate that boys dominate in terms of participation numbers and PA intensity in soccer, basketball and handball.

**Table 3:** Mean proportion of student activity intensity (M%) and standard deviations (SD) and number of observations (n) during recess and lunch break by sex and activity type in 2014 and 2015

Context of Physical Activity observations (via iSOPARC)	Recess & lunch behaviour (VPA%)		Within activity difference (95% CI)	Boys recess & lunch behaviour (VPA%)		Girls recess & lunch behaviour (VPA%)		Between group difference for Boys^+^ and Girls^−^ based on activity type	
	2014 M% (SD) ^n^ (*n* = 290)	2015 M% (SD) ^n^ (*n* = 292)	Mean Difference (p-value)	2014 M% (SD) ^n^ (*n* = 215)	2015 M% (SD) ^n^ (*n* = 245)	2014 M% (SD) ^n^ (*n* = 75)	2015 M% (SD) ^n^ (*n* = 47)	2014 Mean Difference (*p*-value)	2015 Mean Difference (*p*-value)
Aerobics/Dance	41.9 (21.2)^7^	12.6 (16.3)^14^	−29.3 (< 0.01)	N/A	0 (−)^3^	41.9 (21.2)^7^	16.1 (16.8)^11^	N/A	−16.1 (< 0.01)
Jogging/Running	51.2 (27.8) ^53^	27.5 (21.1) ^30^	−23.7 (< 0.01)	45.7 (24.5) ^44^	25.2 (13.1)^20^	77.8 (29.2)^9^	29.4(32.5)^10^	−32.1 (< 0.01)	−4.2 (0.35)
Basketball	36.2 (30.0) ^41^	19.6 (19.0)^19^	−16.6 (< 0.01)	36.6 (29.6)^22^	20.6 (18.9)^18^	35.8 (38.0)^19^	0 (−)^1^	0.8 (0.53)	20.6 (< 0.01)
Rugby/Touch Football	43.6 (28.3) ^71^	56.2 (21.4) ^62^	12.6 (< 0.01)	44.6 (25.8) ^57^	56.2 (21.4) ^62^	39.6 (38.0)^14^	N/A	5.0 (0.67)	N/A
Handball	19.2 (25.8) ^73^	14.0 (22.8) ^84^	−5.2 (0.09)	19.4 (25.9) ^58^	13.8 (21.1) ^71^	18.8 (26.7)^15^	14.9 (31.6)^13^	0.6 (0.53)	−0.9 (0.45)
Soccer	64.4 (26.8)^20^	22.2 (18.6) ^54^	−42.2 (< 0.01)	64.4 (26.8)^20^	24.0 (18.5) ^48^	N/A	8.0 (12.6)^6^	64.4 (< 0.01)	16.0 (0.98)
Racquet games	40.8 (13.7)^8^	8.1 (7.2)^4^	−32.7 (< 0.01)	40.7 (14.8)^7^	8.1 (7.2)^4^	41.0 (−)^1^	N/A	0.3 (0.52)	N/A
Tag/Chasing games	28.6 (21.0)^17^	41.4 (23.9)^25^	12.8 (0.03)	19.0 (20.1)^7^	39.2 (23.2)^19^	35.4 (19.7)^10^	48.6 (26.9)^6^	−16.4 (0.06)	−9.4 (0.15)

^n^= Number of observations in context

Table 4 shows that there were no significant correlations between temperature (*r* = 0.00; *p* = 0.99), school ICSEA (*r* = 0.03; *p* = 0.39), or time of day (r = 0.00; p = 0.99) with student %VPA during recess and lunch breaks (when these were treated as continuous and independent variables).

**Table 4:** Correlations of varying contexts with vigorous physical activity during recess and lunch breaks

	Observations(*n* = 1426)	
	r	*p* value
Environmental factors (PC)		
Temperature	0.00	0.99
School factors (PC)		
School ICSEA	0.03	0.39
Time of day (PC)		
1000 h -1400 h (EDST)	0.00	0.99

*PC* Pearson correlation; *ICSEA* Index of Community Socio-Educational Advantage; *ESDT* Eastern Daylight Savings Time (Australia)

## Discussion

The purpose of this study was to describe the intensity of PA primary school students participate in during recess and lunch breaks in NSW, Australia. Regarding percentage of PA intensity during recess and lunch, our main findings were that students spent the majority of their recess and lunch breaks engaged in sedentary activities. Students only spent around 17% of their recess and lunch breaks engaged in VPA and 37% of their time walking. These findings are comparable to the findings of other playground studies. The SOPLAY(System for Observing Play and Leisure Activity in Youth) [16] data from the Janssen et al.’s (2013) study conducted in the Netherlands [17] reported that primary school children in the control group were engaged in moderate to vigorous physical activity for 38.7% of recess and lunch breaks. In Australia, Willenberg and colleagues^18^ also used SOPLAY, with the percentage of children participating in sedentary, moderate and vigorous activity being 44%, 30% and 27%, respectively, during recess and lunch breaks. These direct observation results are comparable with the behaviour patterns observed in this study.

When examining the data by sex, girls’ spent half the amount of time engaged in VPA (11%) when compared with boys (22%). There was also a gender difference in the percentage of recess and lunch breaks spent in sedentary activities (girls 53%; boys 41%). Whilst conducted in another Australian capital city, Willenberg et al. [18] reported a comparable difference in gender-based %VPA (Boys = 32%; Girls = 22%) and sedentary activities (Boys 39%; Girls 49%) during recess and lunch breaks.

The findings of a discrepancy in the intensity of PA between boys and girls at recess and lunch are clear and worthy of further investigation. Blatchford et el [19] argues that the ‘social’ dynamic of the playground may influence a child’s capacity to engage in more VPA. According to Blatchford and colleagues [19], the school playground is an important social setting, especially for boys. In this study, 582 observations of VPA during recess and lunch breaks, exclusively came from team sport participation (i.e. basketball, rugby/touch, or soccer) (*n* = 267) in which boys participated more frequently and in greater intensity. However, apart from the participation rates, the differences in intensity were only significant in basketball (*p* < 0.01). Future interventions may benefit from investigating the participation in team sports during recess and lunch breaks and providing spaces for these activities to be conducted.

This study also shows that a small number of girls were, at some point, involved in ball games such as rugby/touch football and basketball. However, girls were more likely to engage in tag/chasing games or aerobics/dance activities than boys. These are games where students are less likely to bump into each other or have boys dominate the activity or intensity. So although there were certainly some girls interested in football and basketball, other girls enjoyed playing other kinds of games with less physical collision (and less PA intensity). Given the opportunity, girls may participate in ball games, but might well avoid others that involve physical collisions or boy domination (i.e. football) [19].

Another explanation for the lack of %VPA during recess and lunch by girls may be associated with the requirement for all children at these schools to wear school uniforms. School uniforms have been explained by a small number of quantitative and a larger number of qualitative studies which have shown that having to wear tight, ill-fitting, gender stereotyped or uncomfortable uniforms were major barriers to girls participating in school-based physical activity [20–22] and also may affect physical self-esteem[22]. As such, Dudley and colleagues^20^ recommended that schools policy makers reconsider the daily uniform students are required to wear if they are going to engage with physical education and participate in regular school-based PA.

It has also been proposed in the literature that differences in physical activity participation between boys and girls are more apparent in unstructured setting environments (such as recess and lunch breaks at school) [23]. Our study supports for this finding and therefore suggests that primary school recess and lunch breaks are a particularly important setting for investigating why these discrepancies exist, which is highlighted as we know differences in fundamental movement skills performance and physical activity participation between the sexes widen as they move into adolescence[24]. During primary school skill differences are generally determined by contextual rather than physiological factors[25], hence cultural and environmental factors of a primary school playground may be inhibiting PA intensity of girls compared with boys [26].

Grassed areas were more conducive to %VPA with twice as much %VPA occurring in those areas when compared with hard surfaced areas during recess and lunch breaks. These findings are new and in stark contrast to other Australian playground studies. Willenberg et al.^18^ reported no statistical difference between %VPA on hard and soft surfaces (29%; 27% respectively). These findings indicate that there are perhaps differences in school policy in NSW and Victoria around play in these areas. An explanation for this behaviour pattern could be attributed to play experiences in NSW primary schools being limited for many children due to excessive fear of risk, teacher encouragement or ‘surplus safety’[27] policies compared with Victorian primary schools. There is an increasing body of evidence to suggest safety-related policies regarding school playground behaviour and the role teachers take in enforcing or encouraging behaviour will restrict or enhance the quantity and intensity of PA children undertake during recess and lunch breaks [27].

Contrary to previous research conducted in Australia [12, 18], we found no significant or meaningful relationships between temperature, school socio-economic status, or time of day with the %VPA students engage in during recess and lunch breaks. In terms of temperature, this may be that there was in fact little variation (approximately 10 degrees centigrade) at any given data collection point where previous studies conducted more seasonally based analysis where temperature variation would be greater. To our knowledge, this is the first study that has used the ICSEA scale as a proxy of school socio economic status in an analysis of recess and lunch break physical activity free of intervention. It may be that the lack of sensitivity of ICSEA and iSOPARC to detect specific individual physical activity patterns and their socio-economic status that make this analysis incongruent. Time of day analysis based on this study appears to also be irrelevant.

There are some strengths and limitations associated with the use of the iSOPARC method when measuring intensity of PA in children in schools settings. The first limitation is that iSOPARC requires the observer to conduct scans of the observation area and code the intensity type in quick succession. The dynamic nature of children in a school playground wearing similar uniforms means that the possibility of double coding an individual may occur. This limitation is exacerbated with larger observation areas and more students. A second limitation is the ability of the iSOPARC application to replicate multiple site settings across tablet devices. These currently need to be done individually and thus risk potential data entry error. However, these limitations were offset by the fact that iSOPARC assisted researchers by providing a reliable, efficient and user-friendly means of data collection. Other limitations of the iSOPARC data were its inability to provide accurate estimates of moderate to vigorous PA which make comparisons to some national guidelines difficult. For this reason, we focussed specially on VPA which in turn may be perceived by some as limitation to generalizability. Finally, the inability to control the periods of time spent in recess and lunch breaks made comparable analysis of minutes spent in PA impossible.

## Conclusion

This study shows that recess and lunch breaks are one of the few spaces in which some students can accrue unstructured PA during the school day. The %VPA across both time points was only 16.6% (SD = 23.4) with the majority of the remaining time (46.6%; SD = 30.4) being spent in sedentary activities. Boys are spending up to three times as much time in %VPA compared with girls suggesting that school playgrounds either facilitate or inhibit participation based on sex. %VPA also varied significantly based on the type of surface (*p* < 0.01) and the types of activities the children (*p* < 0.01) were allowed to undertake during recess and lunch.

## Abbreviations

SOPARC: System of Observing Physical Activity in Recreation and Communities (Application version)
ICSEA: Index of Community Socio-Educational Advantage
NSW: New South Wales
PA: Physical activity
SEIFA: Socio-Economic Indexes for Areas
SOPARC: System of Observing Physical Activity in Recreation and Communities
SOPLAY: System for Observing Play and Leisure Activity in Youth
SPSS: Statistical Package for Social Science
VPA: Vigorous physical activity
